# 7.A. Round table: Sustainable health system responses to meet the chronic health care needs of refugees from Ukraine

**DOI:** 10.1093/eurpub/ckac129.403

**Published:** 2022-10-25

**Authors:** Organised by: Centre for Global Chronic Conditions, LSHTM, WHO Europe, Observatory on Health Systems and Policies, Chair persons: Natasha Azzopardi Muscat (WHO Europe), Martin McKee (UK)

## Abstract

**Background:**

Since Russia's reinvasion of Ukraine on 24 February, more than 5 million people have fled the country. Most have gone to the neighbouring countries of Poland, Slovakia, Hungary, Romania, and Moldova. This vulnerable population has significant healthcare needs. Among the most challenging to address will be chronic non-communicable diseases (NCDs), including mental disorders, as these require long-term, continuous care and access to medicines. NCDs are already the biggest contributor to disease burden among Ukrainian adults. About a third have hypertension and 7% have diabetes. Refugees are also at increased risk of mental disorders due to exposure to trauma and ongoing daily stressors. Host country health systems are faced with the challenge of ensuring accessible and affordable care for NCDs and mental disorders to this population. There is no consensus on the most effective and sustainable approaches for achieving this.

**Objectives:**

This workshop will provide a platform for sharing knowledge to support host country health systems. The objectives of the workshop are to i) identify sustainable health system approaches to providing quality health care for NCDs and mental disorders to refugees from Ukraine, and ii) establish research priorities to support host country health system decision-making.

**Format:**

The workshop will consist of a panel with 4 speakers, followed by a roundtable discussion. Each panel member will make a short presentation (5 min) related to health system responses for NCDs and mental disorders in refugee populations. They will highlight challenges faced in the current crisis and evidence of approaches that have worked in other refugee contexts. The roundtable discussion will focus on the adaptation of evidence-based approaches to countries hosting refugees from Ukraine, and the research needed to support this. Interventions will be made by representatives from countries hosting refugees, including from WHO country offices, and from the WHO Europe Migration and Health Programme. All workshop participants will be invited to contribute. The discussion will be chaired by Natasha Azzopardi-Muscat, Director of the Division of Country Health Policies and Systems, WHO European Regional Office. A report on the conclusions of the workshop, including research priorities to support host country health systems, will be published.

**Key messages:**

• Health systems receiving refugees from Ukraine will face challenges in providing them long-term and affordable care for NCDs and mental disorders.

• Drawing on evidence, the workshop will help to identify sustainable health system responses to NCDs and mental disorders, and priorities for research to support host country health systems.

**Introduction**

**Adrianna Murphy**

LSHTM, London, UK

**Impact of the crisis in Ukraine on neighbouring health systems**

**Kayvan Bozorgmehr**

Bielefeld University, Bielefeld, Germany

**Financing to meet the chronic health care needs of refugees**

**Triin Habicht**

Senior Health Economist, WHO Barcelona Office for Health Systems Strengthening, Barcelona, Spain

**Implementing responsive integrated mental health care for refugees**

**Sergiy Bogdanov**

Centre for Mental Health and Psychosocial Support, National University of Kyiv Mohyla Academy, Kyiv, Ukraine

**Ensuring continuous access to NCD treatment among refugees**

**Sigiriya Aebischer Perone**

Geneva University Hospitals, Geneva, Switzerland

